# Benefit of chemotherapy as part of treatment for HPV DNA-positive but p16-negative squamous cell carcinoma of the oropharynx

**DOI:** 10.1038/bjc.2011.542

**Published:** 2011-12-06

**Authors:** E Junor, G Kerr, A Oniscu, S Campbell, I Kouzeli, C Gourley, K Cuschieri

**Affiliations:** 1 Edinburgh Cancer Centre, Western General Hospital, Crewe Road South, Edinburgh EH4 2XU, UK; 2 Department of Pathology, Royal Infirmary of Edinburgh, Edinburgh EH16 4SA, UK; 3 Scottish HPV Reference Laboratory, Royal Infirmary of Edinburgh, Edinburgh EH16 4SA, UK; 4 University of Edinburgh Cancer Research UK Centre, Institute of Genetics and Molecular Medicine, Crewe Road South, Edinburgh EH4 2XR, UK

**Keywords:** HPV, P16, squamous cancer, oropharynx

## Abstract

**Background:**

To determine (a) the cause of an improvement in survival from oropharyngeal squamous cell carcinoma (OSCC) in South East Scotland and (b) whether this improvement was human papillomavirus (HPV) and p16 subtype-dependent.

**Methods:**

Clinicopathological characteristics and outcome data for patients referred with OSCC from 1999 to 2001 (Cohort-1) and 2003 to 2005 (Cohort-2) were obtained. Molecular HPV detection and immunohistochemistry for p16 were performed from paraffin blocks.

**Results:**

Cohort-1 and Cohort-2 contained 118 and 136 patients, respectively. Kaplan–Meier analysis revealed significantly improved survival in Cohort-2 (*P*<0.0001). Sub-classification according to HPV and p16 status revealed no improvement in survival in Class-I (HPV−ve/p16−ve; 47 patients) or Class-III (HPV+ve/p16+ve; 77 patients). However in Class-II (HPV+ve/p16−ve; 56 patients) an increase in 5-year cause-specific survival from 36% in Cohort-1 to 73% in Cohort-2 was detected (*P*=0.0001).

Proportional hazards analysis of 217 patients treated radically demonstrated that significant variables were p16 (*P*<0.0001), N stage (*P*=0.0006) and cohort (*P*=0.0024). Removing cohort from the variables offered to the model showed that, whereas p16 (*P*<0.0001) and N stage (*P*=0.0016) remain significant, chemotherapy (*P*=0.0163) and T stage (*P*=0.0139) are now significant. This suggests that much of the cohort effect is due to the higher use of chemotherapy in the second cohort.

**Conclusion:**

These data suggest that HPV+ve/p16−ve patients constitute a separate subclass of OSCC who may particularly benefit from chemotherapy. They imply that p16 status cannot be considered a surrogate for HPV status, and those trials to de-escalate treatment in HPV+ve OSCC should take p16 status into account.

It is now well established that the incidence of oropharyngeal squamous cell carcinoma (OSCC) has increased over the past 20 years in the United States of America, Europe and Australia (Frisch *et al*, 2000; Nasman *et al*, 2009; Hong *et al*, 2010). This demographic change has been linked to an increase in human papillomavirus (HPV)-associated tumours, particularly in younger, White, non-smoking males (Gillison *et al*, 2008). For example, in Stockholm the percentage of HPV-related squamous cell cancers of the tonsil has increased over the past two decades such that for the period 2006–2007, 93% were HPV+ve. Population-based data from Scotland have shown that the rate of OSCC increased more than any other cancer, with a 2.9-fold increase in men (European Age-Standardised Rate per 100 000 at risk, EASR, 1.96–5.95) and a 2.4-fold increase in women (EASR 0.68–1.54) in the period between 1987 and 2006 (Junor *et al*, 2010). The prevalence/association of HPV in this group has yet to be quantified.

There is a growing evidence base to suggest that HPV+ve OSCC have a better prognosis than HPV−ve tumours, suggesting a clinical utility for HPV testing (Lindel *et al*, 2001; Fakhry *et al*, 2008). Retrospective analysis of HPV status in large randomised clinical trials has shown that the survival of HPV+ve non-smokers was significantly better than HPV+ve smokers and HPV−ve non-smokers, with the HPV−ve smokers having the worst survival (Gillison *et al*, 2009; Ang *et al*, 2010).

Other studies have looked at HPV status, and its association with p16 gene expression and survival (with p16 acting as a surrogate of deregulated early viral gene expression). A consensus has emerged indicating that the best survival is associated with HPV+ve/p16+ve status and worst survival with HPV−ve/p16−ve status (Weinberger *et al*, 2006; Smith *et al*, 2008). However prognosis of the discordant groups (HPV−ve/p16+ve and HPV+ve/p16−ve) is more controversial. p16 has been suggested as a surrogate for HPV positivity (Klaes *et al*, 2002).

In 2002 there was a change in treatment policy for oropharyngeal cancer in the South East Scotland Cancer Research Network. As such survival analysis was performed comparing all patients treated between 1 January 1999 and 31 December 2001 with those treated between 1 January 2003 and 31 December 2005. This revealed a greater than anticipated improvement in survival for the latter cohort. We sought to determine whether the change in survival was driven by the increased prevalence of HPV-associated tumours or the change in treatment.

## Patients and methods

### Patients

The Edinburgh Cancer Centre is the regional referral centre for all patients with head and neck cancer for a population of 1.4 million. 2002 was a year of transition, with a change in patient management policy for patients with squamous cell cancer of the oropharynx treated at the Edinburgh Cancer Centre. A comprehensive database had been collected for all patients diagnosed between 1999 and 2001 as part of a national audit, and a comparative population-based cohort of all patients in South East Scotland diagnosed between 2003 and 2005 was used to determine whether the change in treatment had resulted in any change in survival. Checking against the Scottish Cancer Registry confirms that this study is truly population-based.

Treatment of OSCC prior to 2002 had been preferentially surgery±postoperative radiation or radiation alone. Neck dissection was performed for N2/N3 disease pre-radiotherapy in Cohort-1. Concurrent chemotherapy with radiation became the treatment of choice after 2002. During the period of the later cohort, policy changed to performing a neck dissection after concurrent chemo-irradiation if a complete response had been achieved at the primary site.

Histological diagnosis of SCC of the oropharynx was confirmed by pathology review. Patients who presented with metastatic neck nodes and were only later found to have an oropharyngeal carcinoma were excluded. Patients without paraffin blocks were excluded from the subgroup analysis according to HPV/p16 status but were included in overall survival analysis.

Clinicopathological characteristics and outcome data for all patients were obtained from the electronic database and case notes. Radiotherapy was administered with curative intent over a 4-week schedule in the first cohort and a 6.5-week schedule in the second cohort. A radiobiological correction was made to convert both schedules into 2 Gray Equivalent Dose (EQD) using an *α*/*β* ratio of 10. Intensity-Modulated Radiation Therapy was not used. The chemotherapy regimens used were cisplatin or carboplatin±5-fluorouracil. Methotrexate was used in a small number of patients in the first cohort as part of the UKHAN trial. Patients were followed up by the specialist team.

Ethical approval for this study was obtained from Lothian Research Ethics Committee 2.

### Methods

One representative paraffin block was selected for each case. One 10-*μ*m paraffin section was used for HPV analysis and one 3-*μ*m section was used for p16 immunohistochemical analysis. For molecular HPV detection, nucleic acid extraction was performed by digesting the section for 15 h in proteinase-K (Gilbert *et al*, 2007). The crude lysate was purified using the Qiagen DNA mini kit (Qiagen Ltd, Crawley, UK) according to the manufacturer's instructions. Human papillomavirus amplification and genotyping of extract was performed using the INNO-LiPA HPV Genotyping Extra assay (INNOGENETICS N.V, Gent, Belgium). This is a commercial assay, which involves hybridisation of a short HPV amplicon (generated using SPF10 primers) to a probe array for detection of HPV types, 6, 11, 16, 18, 26, 31, 33, 35, 39, 40, 43, 44, 45, 51, 52, 53, 54, 56, 58, 59, 66, 68, 69/71, 70, 73, 74 and 82. The assay also incorporates detection of the human HLA-DPB1 to check for specimen integrity/eligibility for molecular processing. If a sample tested negative for both the human gene and for the HPV types being tested then it was considered ‘invalid’ for HPV testing (owing to lack of amplifiable human DNA sequence).

Immunohistochemical staining for p16 was performed using the automated Vision BioSystems BOND-maX IHC staining instruments. p16 was detected using the CINtec Histology kit (clone E6H4 provided ready to use without dilution from MTM Laboratories, Heidelberg, Germany) according to the manufacturer's protocol. (Appendix Protocol A2) Negative controls (normal cervix) and positive controls (cervical high-grade intraepithelial neoplasia) were included with each series. Staining was then scored as negative, focal positive and positive on the basis of both nuclear and cytoplasmic staining within the tumour component. Diffuse and continuous cytoplasmic and nuclear staining throughout the tumour cells was considered a positive reaction. A negative reaction was considered in cases where tumour cells were completely negative or staining was identified within the non-dysplastic surface squamous epithelium; benign sub-epithelial sero-mucinous glands; or the cytoplasm of scattered histiocytes, peri-tumoural fibroblasts or follicular dendritic cells within the mucosal associated lymphoid tissue.

### Statistical considerations

The Kaplan–Meier method was used to calculate the actuarial cause-specific survival rates and relapse rates (Kaplan and Meier, 1958). The Mantel–Cox test was used for statistical comparison between curves. Exploratory univariate analysis of survival looked at cohort, age, sex, deprivation category, smoking, drinking, sub-site, grade, stage, T stage, N stage, radical surgery, radical radiotherapy, radiation dose (EQD), radical chemotherapy, p16 and HPV status for radically treated patients. A Cox proportional hazards regression model (PH) was used to assess the independent prognostic significance of these variables. Not all radically treated patients could be included in the proportional hazards analyses owing to incomplete data for some variables. Smoking, drinking, grade and HPV status were removed from consideration as they were the variables with the most incomplete data and were not significant in the preliminary PH analysis. The *P*-value for exclusion of a variable was set at the arbitrary level of 0.2. A significance level of 0.05 was used for the exploratory analysis and 0.01 for any quoted results, unless otherwise specified. Confidence intervals (CIs) are quoted at the 95% level.

## Results

### Patient characteristics

There were 118 patients in Cohort-1 (1999–2001) and 136 in Cohort-2 (2003–2005). Minimum follow-up was 83 and 48 months, respectively. Table 1 summarises the pre-treatment patient/disease characteristics and the treatments administered.

The two cohorts were similar apart from the following: p16 (*P*=0.0243) and HPV (*P*=0.0018) were more often positive in Cohort-2. Surgery was more often the treatment of choice in Cohort-1 (*P*=0.0017), but radiation (*P*=0.0292) and chemotherapy (*P*<0.0001) were more frequently used in Cohort-2 (Table 2).

### Survival

**Figure 1:**
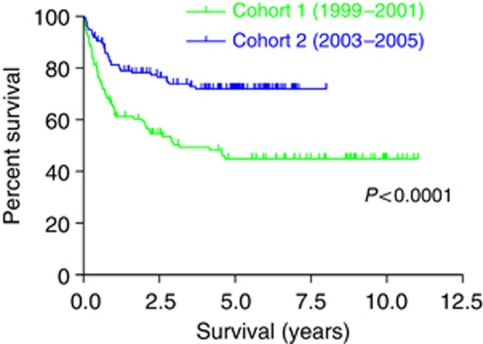
Kaplan–Meier plots illustrating the overall survival of patients in Cohort-1 (1999–2001) and Cohort-2 (2003–2005).

The 2-year cause-specific survival was 59.3% (CI 50.3–68.3%) in Cohort-1 and 78.2% (CI 71.1–85.2%) in Cohort-2. The actuarial estimate was 44.9% (CI 35.4–54.4%) at 5 years in Cohort-1 compared with 72.0% (CI 64.2–79.8%) in Cohort-2. Kaplan–Meier analysis revealed a significant difference in survival between the two cohorts (Figure 1, *P*<0.0001).

Univariate survival analysis revealed p16, HPV, cohort, alcohol and smoking as having a significant effect on cause-specific survival (Table 2). Twenty-five patients received no treatment and eight received only palliative treatment. These 33 patients were excluded from the proportional hazards analysis. The variables age, sex, socio-economic deprivation category (DepCat), cohort, T, N, p16, surgery or not, XRT or not, EQD, and chemotherapy or not were considered, allowing 217 patients (64 events) to be included (Table 3).

Significant variables in order were p16 (HR 0.2 (95% CI 0.107–0.384) *P*<0.0001), nodal status (HR 1.585 (1.218–2.063) *P*=0.0006) and cohort (HR 0.454 (0.273–0.0757) *P*=0.0024). Removing cohort from the variables offered to the model shows that p16 (*P*<0.0001) and nodal status (*P*=0.0016) are still significant. Relaxing the significance level to 0.05% would allow T stage and chemotherapy into the model.

Similarly, offering HPV as a possible prognostic factor with a 0.05% significance would also indicate that it might be of prognostic significance, although in this case only 163 patients (64% of the group under study) are included in the analysis. In the 92 p16+ve patients there were 13 events and no significant prognostic variables. However, if HPV is included (only 80 patients, 12 events) then HPV is significant (*P*<0.0001).

In the 125 p16−ve patients there were 51 events. Significant variables were N stage (*P*=0.0017), chemotherapy (*P*=0.0099) and T stage (*P*=0.0280).

In the HPV+ve patients, only p16 was significant, whereas in the HPV−ve patients, there were no significant variables.

If prognostic group (according to the combination of HPV and p16) is included as a possible prognostic variable instead of HPV and p16 separately, then it is the only significant variable (*P*<0.0001, HR 1.642, CI 1.304–2.067), but only 159 patients and 42 events are analysed.

### Effect of smoking

Cause-specific survival for 1, 2 and 5 years for never smokers was 91.3%, 91.3%, 82.0% for past smokers 90.7%, 83.5%, 67.2% and for current smokers 67.3%, 62.5% and 55.8%.

Smoking was significant in the univariate analysis (*P*<0.0045). Of the 89 p16+ patients for whom smoking status was recorded, 41.6% smoked, 27% were past smokers and 31% were never smokers. In the p16− group 82% were current smokers, 14% past smokers and only 4% were never smokers. Looked at another way 25% of current smokers were p16+, 54% of past smokers were p16+ and 82% of never smokers were p16+.

In the proportional hazard model smoking did not reach statistical significance, but this may have been because p16 was the most significant variable and was strongly associated with smoking.

### Effect of HPV and p16 status on overall survival

**Figure 2:**
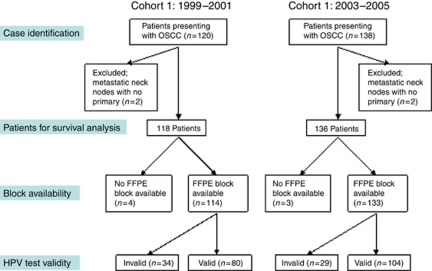
Illustration of case identification and disposition.

A total of 184 patients had valid results on HPV testing, 133 of whom were positive (Figure 2). One hundred and twenty-two were HPV type-16-positive, of whom seven were also positive for another high-risk HPV type. Other subtypes detected without the presence of HPV-16 were HPV-6, 18, 33, 35, 51, 52 and 66. Of these 184 HPV+ve patients, 77 tested positive for p16. Where HPV and p16 were both available, groups were allocated as HPV+ve/p16+ve (77), HPV+ve/p16−ve (56) and HPV−ve/p16−ve (47). There were only four patients who were HPV−ve/p16+ve. Appendix Table A1 shows the sex, age, stage, anatomical sub-site, grade, smoking, drinking, socio-economic deprivation category and treatment for each class by cohort. The second cohort contained more patients who had never smoked, and more patients who were HPV+ve or p16+ve (*P*=0.002 and *P*=0.024, respectively). Treatment in the second cohort was more likely to involve radical radiotherapy (Table 2, *P*=0.03) or chemotherapy (*P*<0.0001), and less likely to have included radical surgery to the primary site. Human papillomavirus (HPV)+ve patients include fewer smokers (*P*=0.004) and are more likely to have had grade-3 tumours (*P*=0.02). p16+ve patients are younger (mean age 56.1 years compared with 62.3 years for p16-negative patients; *P*<0.0001, *t*-test). They are more likely to be male (*P*=0.03), less likely to be smokers (*P*<0.0001) or heavy drinkers (*P*<0.001), more likely to have grade-3 tumours (*P*<0.0001) and to have nodal involvement (*P*=0.002).

p16 and HPV are associated (*P*<0.0001) with 95% of p16+ve patients being HPV+ve, although only 58% of HPV+ve patients were p16+ve.

**Figure 3:**
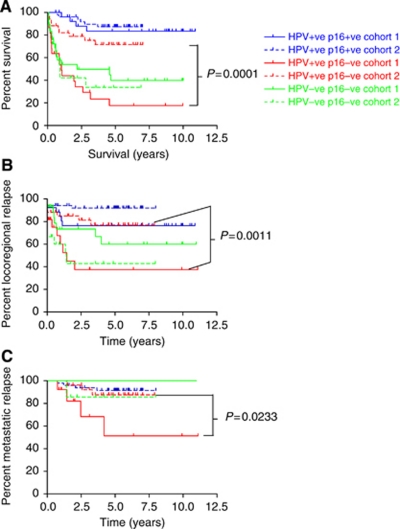
Kaplan–Meier plots illustrating (**A**) the overall survival, (**B**) the frequency of loco-regional relapse and (**C**) the frequency of metastatic relapse of patients in cohorts 1 and 2 according to HPV and p16 status.

Figure 3A shows the survival curves for each of the classes, Class-III (HPV+ve/p16+ve), Class-II (HPV+ve/p16−ve) and Class-I (HPV−ve/p16−ve), by cohort. Survival for classes I and III varies little between the two cohorts. However in Class-II (HPV+ve/p16−ve) 5-year survival rises from 36% in Cohort-1 to 73% in Cohort-2 (*P*=0.0001). When the prognostic features of the HPV+ve/p16−ve groups from cohorts 1 and 2 were compared, the only factor significantly associated with survival was chemotherapy use (*P*=0.0143). All other demographic and treatment differences were not significantly associated with survival (Appendix Table A2).

### Relapse

The 33 patients who were not treated or who received only palliative treatment have been excluded from this analysis. A total of 25 patients (13.1%) failed to achieve a complete response in both primary and nodes (5 were N0) and have been counted as relapsed at time 0. The overall relapse rate was 29.2% (CI 23.1–35.3%) at 2 years and 35.3% (CI 28.7–41.8%) at 5 years. The loco-regional relapse rate was 27.5% (CI 21.5–33.5%) at 2 years and 31.5% (CI 25.2–37.9%) at 5 years. There have been no loco-regional relapses after 4 years.

No complete response was achieved by 6.6% of the 76 HPV+ve/p16+ve patients; 14.9% of the 47 HPV+ve/p16−ve patients; and 22.2% of the 36 HPV−ve/p16−ve patients. Corresponding 2-year loco-regional relapse rates were 13.5%, 28.2% and 43.8%, and 5-year relapse rates 13.5%, 36.2% and 51.8%, respectively.

The rate of loco-regional and metastatic relapse between the cohorts according to HPV and p16 status is shown in Figure 3B and C. Although metastatic relapse is fairly rare, the only significant differences between the cohorts are that both loco-regional (*P*=0.001) and metastatic relapse (*P*=0.023) are more frequent in the earlier cohort in the HPV+ve/p16−ve patients. Combined with the earlier findings this suggests that chemotherapy may have a role in preventing loco-regional and distant relapse, most specifically in the HPV+ve/p16−ve subgroup of patients.

## Discussion

This study was the result of an observation that the disease-specific survival for patients with oropharyngeal squamous cell cancer treated between 2003 and 2005 was significantly better than a similar population treated between 1999 and 2001. The data truly reflect a geographical population but do suffer from all the known problems of retrospective studies. The prevalence of HPV+ve tumours has increased from 67% to 81% in men and from 50% to 85% in women between the two cohorts. The prevalence and rise in HPV+ve tumours in the Scottish population is more in keeping with the reported prevalence in the Stockholm population where 93% of the tonsillar SCC in 2006–2007 were HPV+ve (Nasman *et al*, 2009) than that of the United States of America (72% of oropharynx HPV+ve between 2000 and 2005 (D'Souza *et al*, 2010)) or Australia (60% HPV+ve 2005–2006 (Hong *et al*, 2010)).

The quality of data was good for age, stage, anatomical sub-site, treatment and follow-up, but we were unable to find complete data on smoking and drinking, which limited our analysis. Likewise we were able to retrieve tissue blocks for most patients on which p16 analysis could be performed. Human papillomavirus status could not be determined for approximately 30% of the cases owing to specimen quality. In these cases dilution of template DNA did not improve amplification (data not shown). Degradation of nucleic acid from paraffin-embedded blocks, particularly those stored in excess of 5 years, is well documented (Gilbert *et al*, 2007).

p16 was the most significant prognostic variable, which concurs with other studies.

The effect of smoking reported in other major studies (Ang *et al*, 2010) did not appear as a significant variable in the multivariate analysis, but this may have been due to the very strong association between p16 status and smoking. Interestingly, 75% of smokers were p16− and 82% of non smokers were p16+. When Kaplan–Meier survival curves are created for the study population as a whole, survival for past smokers lies mid-way between non-smokers and current smokers.

The striking finding in this study was that the improvement in survival between the first and second cohorts appeared because of improved outcome in the HPV+ve/p16−ve patients with a 5-year cause-specific survival of 36% in the earlier cohort compared with 73% in the later cohort (*P*=0.0001). As reported by other investigators, HPV+ve/p16+ve patients had excellent survival and HPV−ve/p16−ve tumours had the worst survival (Smith *et al*, 2008). The survival difference was not easily explained by smoking and excessive alcohol intake, as if anything the second cohort contained more current smokers and heavy drinkers. The major difference in treatment between the two cohorts in Class-II was a greater use of chemotherapy in addition to radiation (19 out of 31 (61.2%) in the later cohort compared with 5 out of 16 (31.2%) in the earlier cohort).

We believe these results indicate that the HPV+ve/p16−ve patients are a distinct group who particularly benefit from the use of chemotherapy in addition to radiation.

Comparing the two cohorts did show that the later cohort contained a higher proportion of HPV+ve tumours and that there were more non-smokers in the second cohort. The increase in HPV positivity and number of non-smokers in the second cohort was similar for men and women. Multivariate analysis suggested that the difference in survival was therefore caused by a change in treatment (predominantly to an increase in the use of chemotherapy) and a difference in T stage. This is in agreement with a major meta-analysis of randomised trials showing that, in the primary setting, chemotherapy in addition to radiation compared with radiation alone was beneficial (Pignon *et al*, 2000).

We were unable to address the addition of chemotherapy to radiation in the post-operative setting as only one patient received this treatment. Weinberger was the first to report a ‘three-class hypothesis’ (Weinberger *et al*, 2006). He called (HPV+ve/p16+ve) HPV-active Class-III 18 out of 78; (HPV+ve/p16−ve) HPV-inactive Class-II 29 out of 78; and (HPV−ve/p16−ve) HPV-negative Class-I 30 out of 78. All the patients included in their study had received either radiation alone or surgery with post-operative radiation. In Weinberger's study patients in Class-III had 5-year DFS of 75% *vs* 15% for patients with Class-I and 13% for patients with Class-II. Interestingly the local recurrence rate was higher in Class-II (74%) than in Class-I (45%) and Class-III (14%). Our 5-year local recurrence rates were 13.5% for Class-III, 36.2% for Class-II and 51.8% for Class-I. The increased percentage of our patients falling into Class-III likely reflects not only a higher prevalence of HPV+ve tumours in our population, but also the time period covered by the two studies, 1980–99 in Weinberger's study and 1999–2005 for ours. The poor outcome of the Class-II patients in Weinberg's study (the vast majority of whom received no chemotherapy) mirrors the outcome of our Cohort-1 patients, adding weight to the suggestion that the improved outcome seen in the Class-II patients in Cohort-2 of our study was caused by the increased use of chemotherapy.

Controversy exists as to whether the HPV-inactive Class-II (HPV+ve/p16−ve) exists as a discrete clinical entity. It is interesting that in a recent study of 239 cases, Lewis *et al* (2010) found only five to be HPV+ve/p16−ve using DNA ISH. A conclusion of this study was that p16 testing alone would be sufficient for delineation of meaningful clinical categories, but importantly PCR was not performed on the p16−ve cases. Our data would not only strongly support the existence of this separate molecular group, but would also indicate an important role for the use of chemotherapy in this subgroup.

It is possible that our assignment of patients to HPV/p16 classes could be affected by technical issues such as false positivity of the HPV assay in the HPV+ve/p16−ve tumours. If this was the case then the true assignment of this class would be HPV−ve/p16−ve and this is unlikely as the survival of our HPV+ve/p16−ve patients in Cohort-2 was much closer to that of HPV+ve/p16+ve patients than it was to that of HPV−ve/p16−ve patients. The different behaviour is in itself suggestive that the HPV+ve/p16−ve class is a discrete clinical entity.

In terms of a mechanism behind our observations, the HPV-encoded oncoproteins E6 and E7 are responsible for HPV-associated tumorigenesis in oropharyngeal and other cancers. E6 causes degradation of p53 through ubiquitin-mediated proteolysis (Wiest *et al*, 2002). As such, E6-expressing cells are not capable of a normal p53 response and show features of genomic instability (Duensing and Munger, 2004). E7 binds to and inactivates pRb, causing the cell to enter S-phase, resulting in cell-cycle disruption, proliferation and malignant transformation. Inactivation of pRb also results in upregulation of p16 (Wiest *et al*, 2002). The combined effect of E6 and E7 on the p53 and pRb pathways, respectively, while resulting in tumorigenesis may both contribute to the high radiosensitivity and chemosensitivity of HPV+ve/p16+ve tumours reported here and elsewhere. It is possible that either through abrogation of the effect of E7 or as a result of epigenetic or mutational silencing of host p16 (O'Regan *et al*, 2008), the HPV+ve/p16−ve tumours lack the molecular consequences of E7 expression and that one of the consequences of this is lower radiosensitivity compared with HPV+ve/p16+ve tumours. It could be argued that chemotherapy with agents such as platinum, to which tumours with genomic instability are recognised to be sensitive, is necessary to optimise the outcome in these patients.

This study confirms the excellent prognosis of HPV+ve/p16+ve patients and lends evidence to the suggestion of de-escalation of treatment trials in this group.

It identifies the HPV+ve/p16−ve group as a clinically distinct entity and strongly supports the use of chemotherapy in addition to radiation in this group. As this was a retrospective study we would strongly propose a clinical trial to address this specific issue and that further trials using novel strategies should be considered in the HPV−ve/p16−ve group.

## Figures and Tables

**Table 1:** Patient demographics and clinical characteristics

	**Total**		**1999–2001**		**2003–2005**	
**Characteristic**	**No.**	**%**	**No.**	**%**	**No.**	**%**
No. of cases	254	100	118	46	136	54
						
*Sex*						
Male	182	72	85	72	97	71
Female	72	28	33	28	39	29
						
*Age (years)*						
Mean	60		60		60	
						
*Disease site*						
Tonsil	119	47	63	53	56	41
Soft palate	31	12	10	8	21	15
Base of tongue	73	29	34	29	39	29
Oropharynx, NOS	31	12	11	9	20	15
						
*Staging*						
T1–2	124	49	63	53	61	45
T3–4	130	51	55	47	75	55
N0	77	30	37	31	40	29
N1–3	176	69	81	69	95	70
N*x*	1	0	0	0	1	1
Stage I–II	49	19	27	23	22	16
Stage III–IV	205	81	91	77	114	84
						
*P16*						
Positive	94	37	35	30	59	43
Negative	153	60	79	67	74	54
NK	7	3	4	3	3	2
						
*HPV*						
Positive	133	52	48	41	85	63
Negative	51	20	32	27	19	14
Invalid	63	25	34	29	29	21
NK	7	3	4	3	3	2
						
*Treatment*						
None	25	10	16	14	9	7
Palliative chemo	6	2	4	3	2	1
Surgery alone^a^	12	5	6	5	6	4
Surgery+PORT	22	9	19	16	3	2
Surg, POCRT	3	1	1	1	2	1
XRT alone	64	25	43	36	21	15
Chemo and XRT	19	7	1	1	18	13
Concom chemo/XRT	103	41	28	24	75	56
						
*Smoking*						
Never	35	14	11	9	24	18
Ex	44	17	16	14	28	21
Current	158	62	78	66	80	59
NK	17	7	13	11	4	3
						
*Drinking*						
Never	13	5	5	4	8	6
Previous	20	8	6	5	14	10
Social	102	40	47	40	55	40
Excess	99	39	45	38	54	40
NK	20	8	15	13	5	4

Abbreviations: Chemo and XRT=neoadjuvant chemotherapy followed by radiation alone; Concom=concomitant chemotherapy and radiation; POCRT=postoperative chemoradiation; PORT=postoperative radiation; Surg, XRT+chemo=surgery, postoperative concomitant chemotherapy and radiation.

a Patients counted as having surgery alone are those treated with radical surgery for the primary tumour, not those solely undergoing neck dissection.

**Table 2:** *P*-values for associations between variables and univariate survival analysis

	**Associations (significant *P* from *χ*^2^-test)**			***P***
	**Cohort**	**HPV**	**p16**	**Univariate CSSR**
Cohort		0.0018	0.0243	0.0006
Sex			0.0314	0.1781
Age			0.0048	0.0646
Smoking	0.0774	0.0018	<0.0001	0.045
Drinking		0.0783	<0.0001	0.0127
DepCat				0.2430
T				0.2057
N			0.0016	0.5815
Stage			0.0054	0.3303
Grade		0.0176	<0.0001	0.1873
p16	0.0243	<0.0001		<0.0001 (1)
HPV	0.0018		<0.0001	<0.0001 (2)
Surgery	0.0017			0.9507
XRT	0.0292	0.0113	0.0002	0.3538
Chemo	<0.0001	0.0181	<0.0001	0.1954

Abbreviations: CSSR=cause-specific survival rate; DepCat=socioeconomic deprivation category. (1) test statistic 35.0; (2) test statistic 24.0.

**Table 3:** Results of PH analysis

**Variable**	***P***	**Hazards ratio**	**CI for hazards ratio**
p16	<0.0001	0.211	0.111–0.398
N stage	0.0011	1.551	1.192–2.017
Cohort	0.0037	0.471	0.283–0.0783
			
*Excluding cohort*			
p16	<0.0001	0.20	0.106–0.377
N stage	0.0034	1.471	1.136–1.903
T stage	0.0139	1.366	1.065–1.752
Chemotherapy	0.0163	0.464	0.248–0.868

**Table A1:** Patient demographics and clinical characteristics according to HPV and p16 status

	**HPV+ve, p16+ve**		**HPV+ve, p16−ve**		**HPV−ve, p16−ve**	
	**99–01**	**03–05**	**99–01**	**03–05**	**99–01**	**03–05**
Total referrals	26	51	22	34	29	18
						
*Sex*						
Male	21	38	16	18	18	13
Female	5	13	6	16	11	5
						
*Mean age*	54.5	55.6	63.3	60.1	59.3	66.1
Male	54.0	54.7	61.3	58.7	64.4	66.7
Female	56.2	58.4	68.5	61.6	51.6	64.4
						
*Sub-site*						
Tonsil	17	33	12	10	12	3
Palate	2	0	1	8	2	4
Base of tongue	5	16	7	7	12	6
Oropharynx, NOS	2	2	2	9	3	5
						
*T*						
T1	10	13	6	13	8	2
T2	5	16	3	5	7	5
T3	4	12	4	5	5	3
T4	7	10	9	11	9	8
						
*N*						
N0	5	8	6	14	8	7
N1	4	5	5	6	5	2
N2	14	33	9	12	13	8
N3	3	5	2	1	3	1
N*X*	0	0	0	1	0	0
						
*Stage*						
I	2	4	2	8	6	1
II	0	1	1	2	1	3
III	5	6	4	6	5	4
IV	19	40	15	18	17	10
						
*Grade*						
1	0	2	2	2	3	0
2	9	8	4	13	15	5
3	13	25	5	6	2	6
*X*	4	16	11	13	9	7
						
*Smoking*						
Never	6	18	2	1	0	2
Ex	7	16	2	5	2	3
Current	13	15	16	26	24	13
NK	0	2	2	2	3	0
						
*Drinking*						
Never	3	3	0	3	0	1
Previous excess	1	4	0	4	1	1
Social	12	34	10	7	12	3
Excess	10	8	9	18	12	12
NK	0	2	3	2	4	1
						
*DepCat*						
1	1	4	1	1	0	1
2	2	10	1	5	3	2
3	11	11	5	8	7	3
4	7	18	9	7	8	6
5	4	7	3	7	9	4
6	1	0	2	4	1	1
7	0	1	1	2	1	1
						
*Treatment*						
Surgery	12	3	3	3	4	2
Rad XRT	24	49	16	28	19	14
Rad chemo	10	44	5	19	6	11
Surgery alone	2	1	0	3	2	1
Surgery+PORT	10	1	2	0	2	0
Surg POCRT	0	1	1	0	0	1
XRT alone	4	4	9	9	11	3
Concom chemo/RT	10	34	4	15	6	8
Chemo and XRT	0	9	0	4	0	2
No radical treatment	0	1	6	3	8	3

Abbreviations: CTXRT=concomitant chemotherapy and radiation; CXCT=neoadjuvant chemotherapy followed by radiation; DepCat=socioeconomic deprivation category; Rad Chemo=concomitant chemotherapy and radiation; Rad XRT=single-modality radiation with curative intent; SCXRT=surgery with postoperative chemotherapy and radiation; SU=surgery; SUXRT=surgery with postoperative radiation; XRT=radiation alone.

**Table A2:** Patient demographics and clinical characteristics for HPV+ve/p16–ve patients

	**99–01**	**03–05**	***P*-value**
Total referrals	22	34	
			
*Sex*			
Male	16	18	0.139
Female	6	16	
			
*Mean age*	63.3	60.1	0.319
Male	61.3	58.7	0.511
Female	68.5	61.6	0.118
			
*Sub-site*			
Tonsil	12	10	0.049
Palate	1	8	
Base of tongue	7	7	
Oropharynx, NOS	2	9	
			
*T*			
T1	6	13	0.836
T2	3	5	
T3	4	5	
T4	9	11	
			
*N*			
N0	6	14	0.587
N1	5	6	
N2	9	12	
N3	2	1	
N*X*	0	1	
			
*Stage*			
I	2	8	0.545
II	1	2	
III	4	6	
IV	15	18	
			
*Grade*			
1	2	2	0.383
2	4	13	
3	5	6	
*X*	11	13	
			
*Smoking*			
Never	2	1	0.522
Ex	2	5	
Current	16	26	
NK	2	2	
			
*Drinking*			
Never	0	3	0.052
Previous excess	0	4	
Social	10	7	
Excess	9	18	
NK	3	2	
			
*DepCat*			
1	1	1	0.702
2	1	5	
3	5	8	
4	9	7	
5	3	7	
6	2	4	
7	1	2	
			
*Treatment*			
Surgery	3	3	0.570
Rad XRT	16	28	0.391
Rad chemo	5	19	0.014
Surgery alone	0	3	0.027
Surgery+PORT	2	0	
Surg POCRT	1	0	
XRT alone	9	9	
Concom chemo/RT	4	15	
Chemo and XRT	0	4	
No radical treatment	6	3	

Abbreviations: CTXRT=concomitant chemotherapy and radiation; CXCT=neoadjuvant chemotherapy followed by radiation; DepCat=socioeconomic socioeconomic deprivation category; Rad Chemo=concomitant chemotherapy and radiation; RAD XRT=single-modality radiation with curative intent; SCXRT=surgery with postoperative chemotherapy and radiation; SU=surgery; SUXRT=surgery with postoperative radiation; XRT=radiation alone.

## Appendix Protocol A2

Protocol for retrieval of antigen and antibody staining according to the CINtec Histology kit.

Staining for p16 was performed using the CINtec Histology Kit from MTM Laboratories (Heidelberg, Germany).

The kit contains all the necessary reagents for immunohistochemical detection of the p16INK4a antigen. The kit is designed to be used on paraffin-embedded tissue specimens. The staining was performed on a BOND-maX automated stainer in an accredited pathology lab and according to the manufacturer's protocol.

The following steps were performed:

1. De-paraffinisation in xylene and rehydration in alcohol solutions (ethanol 95% and 70%).
2. Epitope retrieval in a water bath at 95–99 °C for 10 min.
3. Cool-down in epitope retrieval solution at room temperature (20–25 °C) for 20 min.
4. Rinse with wash buffer.
5. Quenching of endogenous peroxidase with a peroxidase-blocking reagent (3% hydrogen peroxide) for 5 min.
6. Rinse with wash buffer.
7. Application of primary antibody – mouse anti-human p16 antibody – for 30 min and negative controls in parallel (please see below).
8. Rinse with wash buffer.
9. Application of visualisation reagent (a polymer reagent conjugated with horseradish peroxidase and affinity-purified goat anti-mouse Fab′ fragments) for 30 min.
10. Rinse with wash buffer three times.
11. Application of DAB-buffered substrate for 10 min.
12. Rinse with wash buffer.
13. Rinse with deionised water.
14. Counterstaining with haematoxylin and mounting of slides with permanent mounting media.

The p16 antibody, which is ready diluted, is a mouse anti-human monoclonal antibody (clone E6H4 from MTM Laboratories, Heidelberg, Germany). The negative control also ready to use is a mouse anti-rat oxytocin-related neurophysin antibody (rat oxytocin-related neurophysin is not present in human tissues).

Table A1

Table A2
